# Herpes zoster ophthalmicus presenting with orbital myositis prior to the appearance of vesicular lesions: A case report and mini‑review of the literature

**DOI:** 10.3892/mi.2024.185

**Published:** 2024-08-02

**Authors:** Jamir Pitton Rissardo, Pranav Patel, Ana Leticia Fornari Caprara

**Affiliations:** 1 Department of Neurology, Cooper University Hospital, Camden, NJ 08103, USA; 2 Cooper Medical School of Rowan University, Camden, NJ 08103, USA

**Keywords:** herpes zoster, varicella zoster, varicella-zoster virus, orbital syndrome, orbital myositis, herpes zoster ophthalmicus, shingles, immunocompetent

## Abstract

All orbital tissues, including extra-ocular muscles, can be affected by the varicella-zoster virus (VZV). However, only a minority of all individuals with herpes zoster infections present with herpes zoster ophthalmicus. The present study reports the case of a middle-aged male patient presenting with an acute intractable right-sided headache. His neurological examination yielded normal results. The analysis of cerebrospinal fluid by biochemistry and cultural analysis yielded normal results; however, the analysis of this fluid using polymerase chain reaction yielded a positive result for VZV. Thus, treatment with acyclovir was commenced. Brain magnetic resonance imaging revealed a bilateral intraorbital intraconal enhancement consistent with myositis. His symptoms evolved into a shock-like pain over the scalp associated with painful ocular movements. On the 2nd day of admission, he developed new vesicular lesions found on the right-side cranial nerve V1 dermatome. By the 6th day of admission, he was asymptomatic, and his physical examination revealed the resolution of the dermatologic manifestations of the VZV. The patient was stable for outpatient follow-up with ophthalmology and was discharged on an oral valacyclovir course for 7 days. To the authors' knowledge, there are four cases reported in the literature of herpes zoster ophthalmicus with orbital myositis prior to the appearance of vesicular lesions. Thus, it is suggested that VZV serology be investigated before a final diagnosis of idiopathic orbital myositis is made.

## Introduction

All orbital tissues, including extra-ocular muscles, can be affected by the varicella-zoster virus (VZV) (1). However, <10% of all patients with VZV infections present with herpes zoster ophthalmicus. Notably, there is a great variability as regards orbital involvement, affecting 20-70% of individuals with herpes zoster ophthalmicus (2). Usually, ophthalmic complications of herpes zoster opthalmicus occur between 5 and 14 days following cutaneous lesions (3).

The present study reports the case of a middle-aged male patient presenting with orbital myositis due to herpes zoster ophthalmicus prior to the appearance of vesicular lesions. To the authors' knowledge, there are only four cases reported in the literature of herpes zoster ophthalmicus with orbital myositis prior to the appearance of vesicular lesions (4-7).

## Case report

A 56-year-old male patient with a prior medical history of hyperlipidemia, and who was not on pharmacotherapy, arrived at the Emergency Department of Cooper University Hospital, Camden, NJ, USA with an acute intractable right-sided headache. His headache began 3 days prior to his presentation and was described as primarily right-sided with radiation to the neck, along with associated fevers, blurred vision and photophobia. He stated he was cleaning out an old farm devoid of animals during the onset of the headache. Of note, the patient had no prior history of headaches, and his only medication has been testosterone cypionate by intramuscular route once every 4 weeks over the past 6 months. He had no previous surgical history and no pertinent family history of neuropsychiatric disorders.

In the emergency department, an examination of his vital signs revealed a temperature of 98.5 F, a blood pressure of 141/67 mmHg, a heart rate of 88 beats per minute and an oxygen saturation of 96% in room air. A cardiopulmonary examination did not reveal any notable findings. A head examination revealed normocephalic and atraumatic findings, with normal bilateral external ear canals. His neurological examination yielded normal results. His sensory and motor examination revealed no focal deficits and his coordination was normal. There was no evidence of skin changes. In consideration of a differential inclusive of intracranial hemorrhage vs. meningitis vs. venous thrombosis secondary to anabolic abuse, a thorough workup was performed, including a cranial computed tomography (CT) scan, cranial CT-venogram and a lumbar puncture.

**Figure 1:**
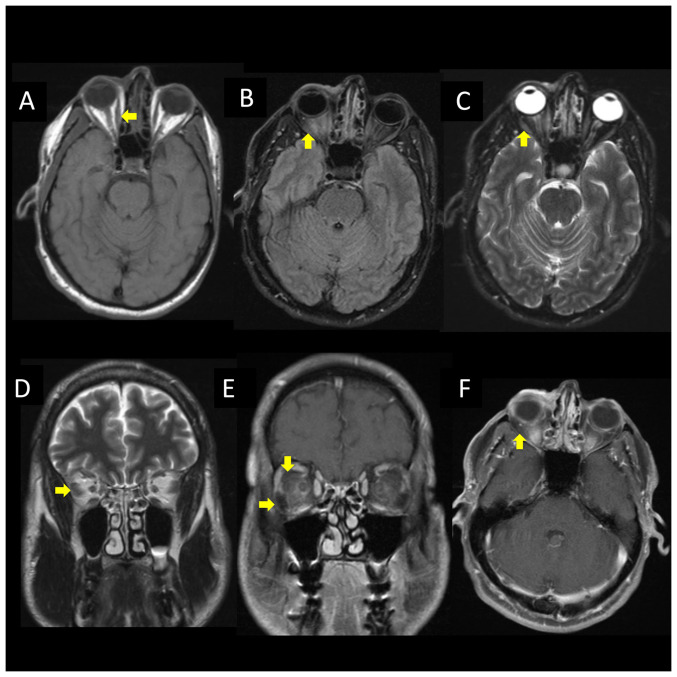
Brain magnetic resonance imaging illustrating myositis of the right extraocular muscles. Patchy bilateral intraorbital intraconal enhancement around the optic nerve sheath complexes and asymmetric thickening and enhancement of the right extraocular muscles. (A) Axial T1-weighted, (B) fluid attenuated inversion recovery, (C) T2-weighted fat suppressed, and (F) T1-weighted turbo spin echo images; (D) coronal T2-weighted and (E) T1-weighted turbo spin echo images are shown.

The cranial CT and CT venogram scans did not reveal any notable findings. The lumbar puncture demonstrated a protein level of 38 mg/dl, glucose level of 65 mg/dl and an acellular fluid analysis. However, polymerase chain reaction of the cerebrospinal fluid (CSF) revealed a positive result for VZV. The patient was thus commenced on intravenous treatment with acyclovir at 10 mg/kg every 8 h, and a follow-up brain magnetic resonance imaging was performed, revealing bilateral intraorbital intraconal enhancement consistent with herpes zoster ophthalmicus with myositis (Fig. 1). An infectious disease consult was pursued, and a course of intravenous acyclovir was continued throughout his hospitalization period. The results of the complete assessment of all the laboratory tests performed are presented in Table I.

**Figure 2:**
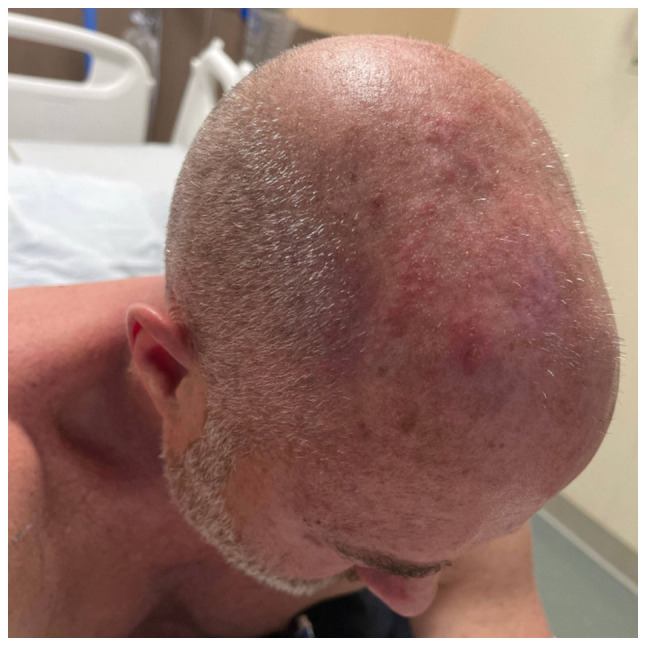
Anatomical view of the forehead showing vesicular lesions on the 2nd day of admission. The lesions crusted within 2 weeks at the follow-up and disappeared within 1 month.

During his admission, the symptoms of the patient evolved into a shock-like pain over the scalp associated with pain in ocular movements. His clinical course was further complicated by a follicular reaction on the palpebral conjunctiva, which was determined to be a viral follicular conjunctivitis likely secondary to the VZV. On the 2nd day of admission, he developed new vesicular lesions found on the right-side cranial nerve V1 dermatome (Fig. 2). On the 4th day of admission, he began to experience relief from his headache and ocular disturbances. By the 6th day of admission, he had experienced the complete resolution of his symptoms, and his physical examination revealed the resolution of the dermatologic manifestations of the VZV. The culture of his CSF remained negative. The patient was stable for outpatient follow-up with ophthalmology and was discharged on an oral valacyclovir (1 g, 2 tablets every 6 h) course for 7 days.

## Discussion

Orbital myositis is characterized by worsening pain with eye movements. Other common features in individuals with orbital myositis are proptosis, swollen eyelids and hyperemic conjunctiva. Common causes of orbital myositis are thyroid disease, syphilis and auto-immune diseases, which were not observed in the patient described herein. His ocular pain related to eye movements significantly improved after treatment with acyclovir was commenced, and he fully recovered within 1 week of therapy.

The patient was treated with acyclovir intravenously when he was hospitalized and his treatment regiment was then changed to oral valacyclovir for a period of 7 days. As clinical signs and symptoms associated with VZV, the CNS infection improved and the patient was able to take oral medications; therapy was thus completed with oral treatment. Acyclovir and valacyclovir are both effective against VZV CNS infections. Notably, acyclovir, compared to valacyclovir, has some drawbacks, including dosing at multiple times per day, poor oral bioavailability and inadequate CNS penetration/levels, which render oral acyclovir a suboptimal option for such cases (8).

A previous study by Marsh and Cooper (9), using a large cohort, assessed 1,356 patients with herpes zoster ophthalmicus. That study found that extraocular muscle involvement was associated with the appearance of skin lesions and that the severity of the rash was related to recurrence and postherpetic neuralgia. However, that study did not describe neuroimaging analyses (9). In addition, to date, to the best of our knowledge, there are no radiological and histopathological studies available associating orbital myositis with herpes zoster ophthalmicus.

In 1948, Parkinson (10) described a case of herpes zoster ophthalmicus followed by ptosis on the same side. He reported that idiopathic cases of myositis should be reserved only after the availability of negative serological and immunological results for herpes zoster (10). Of note, 10 years later, Lewis (11) reported a syndrome known as ‘ophthalmic zoster sine herpete’, characterized by orbital pain, extraocular palsy and periorbital skin swelling without skin rashes.

In the case in the present study, the patient developed a significant headache with concern from vascular etiology. Following initial neuroimaging, the CSF was analyzed, and the VZV infection was noticed. A possible explanation for the headache could be related to prodromal symptoms or myositis affecting the extraocular muscles. Ophthalmic complications following herpes zoster ophthalmicus may result from inflammatory changes, nerve damage, or secondary tissue scarring.

The authors searched six databases to locate studies describing the appearance of myositis prior to skin manifestations in individuals with herpes zoster ophthalmicus published from 1991 to January, 2024 in electronic form. The Excerpta Medica (Embase), Google Scholar, Latin American and Caribbean Health Sciences Literature (Lilacs), Medline, Scientific Electronic Library Online (SciELO), and ScienceDirect databases were searched. Publications in the English language were included in the search (Table II).

To the authors' knowledge, Volpe and Shore (4) described the first case of orbital myositis appearing before skin manifestations in individuals with herpes zoster ophthalmicus. They reported the enlargement of extraocular muscles with tendon sparing on a cranial computed tomography scan, which appeared one day before the appearance of the skin rashes.

Oh *et al* (12) reported a patient presenting with eyelid swelling and headache; that study is not included in Table II. They described it as rare, as they considered only vesicular rashes as a skin manifestation (12). Nevertheless, that case illustrates a common presentation of individuals with herpes zoster with skin lesions appearing prior to orbital myositis (8). In addition, Park and Lee (13), Chiang *et al* (14), Bae and An (15) and Pereira *et al* (16) and the first patient reported by Bak *et al* (7) developed periorbital erythematous edema at presentation; these studies are not included in Table II.

The general rule is that the optimal time for initiating antiviral medication is within 72 h following the onset of the vesicular rash. However, the role of systemic steroid therapy in acute orbital syndromes caused by herpes zoster ophthalmicus needs to be further investigated. In the case described herein, this class of medication was not prescribed. However, corticosteroids can attenuate the pain, and may also influence the course of the viral infection by inflammatory pathways.

A possible differential diagnosis for the case in the present study is testosterone-induced myositis. The patient had used testosterone for the past 6 months due to low testosterone levels and symptoms of sexual dysfunction, mood disorder and generalized weakness. Notably, testosterone-induced orbital myositis is uncommonly observed in humans, although it is a common finding in rat models (17). However, myositis secondary to testosterone was considered unlikely in the patient in the present study, as the patient had used testosterone for a long period of tims and had VZV found in the CSF.

In conclusion, patients with orbital pain who have a significant headache should be further investigated. Herpes zoster virus serology needs to be investigated before a final diagnosis of idiopathic orbital myositis is made. The case described in the present study favors management with oral antivirals following a course of at least 5 days with intravenous medications.

## Figures and Tables

**Table I:** Laboratory tests performed for the patent in the present study.

Laboratory tests	Results
Complete blood cell count	White blood cells (5.41; 10x3/µl), red blood cells (5.7 10x6/µl), hemoglobin (16.9 g/dl), hematocrit (49.50%), mean corpuscular volume (86.8 fl), mean corpuscular hemoglobin (29.6 pg), mean corpuscular hemoglobin concentration (34.1 g/dl), red cell distribution width (12.80%), platelet count (212; 10x3/µl), basophils (0.40%), eosinophils (1.50%), granulocytes (53.50%), lymphocytes (29.20%) and monocytes (14.8%)
Basic metabolic panel	Glucose (83 mg/dl), blood urea nitrogen (12 mg/dl), creatinine (1.32 mg/dl), sodium (141 mmol/l), potassium (5.1 mmol/l), chloride (105 mmol/l), CO_2_ (24 mmol/l), calcium (9.2 mg/dl), eGFR [63 ml/min/(1.73_m^2^)]
HIV antibody	Non-reactive
HIV p24 antigen	Non-reactive
COVID-19 PCR	Negative
Influenza A	Negative
Influenza B	Negative
Lyme antibody	Negative
Cerebrospinal fluid analysis	Appearance (clear and colorless), protein (38 mg/dl), corrected nucleated cells (2/µl), red blood cells (<1/µl), glucose (65 mg/dl), cryptococcal antigen (negative)
Cerebrospinal fluid polymerase chain reaction	*Escherichia coli* (negative), *Neisseria meningitidis* (negative), *Haemophilus* *influenzae* (negative), *Listeria monocytogenes* (negative), *Group B* *streptococcus* (negative), *Streptococcus pneumoniae* (negative), *Cryptococcus* *neoformans* (negative), *Cytomegalovirus* (negative), *Herpes simplex virus* 1 and 2 (negative), *Enterovirus* (negative), varicella zoster virus (positive)
Cerebrospinal fluid cultures	Acid-fast bacteria (no growth), cerebrospinal fluid fungal (no growth), cerebrospinal fluid (no growth)

eGFR, estimated glomerular filtration rate.

**Table II:** Cases reported in the literature of orbital myositis preceding skin symptoms in patients with varicella zoster infection.

Author(s), year of publication	Age (years)/sex	Comorbidities	Time^a^ (days)	Neurologic manifestations	Management and outcomes	Comments	(Refs.)
Volpe and Shore, 1991	45/M	Colitis	3	Proptosis	Initially, oral prednisone for idiopathic orbital inflammation was prescribed. After the skin lesion appeared, oral acyclovir and prednisone for three days were prescribed. After three days, the symptoms returned, and he received seven days of the same therapy. A complete imaging resolution was observed within seven weeks.	First report. Only a cranial CT scan was performed.	(4)
Kawasaki and Borruat, 2003	47/F	NA	4	Proptosis and pain worsen with ocular movements	IV and topical acyclovir, and. prednisone	First report with. brain MRI	(5)
Tseng, 2008	54/M	Liver cirrhosis	7	Proptosis and pain worsen with ocular movements	7 Days of IV acyclovir. treatment and discharged. After 7 days, he returned with the same symptoms, and he received 7 days of valacyclovir. A complete imaging resolution was observed within 3 months. PHN was observed at the follow-up	Visual evoked potential was normal. Hutchinson's sign.	(6)
Bak *et al*, 2018	59/F	Healthy	8	Proptosis	IV dexamethasone 10 mg once a day for 2 days and oral famciclovir 750 mg once a day for 10 days were administered. Oral prednisolone 10 mg once a day for 10 days was prescribed.	Dacryoadenitis.	(7)
Rissardo *et al*	56/M	Hyperlipidemia	5	Pain worsening with ocular movements	IV acyclovir 10 mg/kg every 8 h for 6 days, followed by oral valacyclovir course for 7 days.		Present study

^a^Time indicates the time between the presentation of the initial symptoms and the appearance of skin lesions. M, male; F, female; IV, intravenous; MRI, magnetic resonance imaging; PHN, postherpetic neuralgia.

## Data Availability

The datasets used and/or analyzed during the current study are available from the corresponding author on reasonable request.
